# Metastasis-associated lung adenocarcinoma transcript 1 overexpression in testis contributes to idiopathic non-obstructive azoospermia via repressing ETS variant transcription factor 5

**DOI:** 10.1186/s43556-024-00235-6

**Published:** 2024-12-17

**Authors:** Lei Wei, Zonggang Feng, Qian Dou, Pengfen Li, Xinghua Zhao, Bin Hao

**Affiliations:** 1 Reproductive medical center of The Second Affiliated Hospital of Zhengzhou University, No. 2 Jingba Road, Zhengzhou, Henan 450014 China; 2 Department of Urology, The Second Affiliated Hospital of Zhengzhou University, No. 2 Jingba Road, Zhengzhou, Henan 450014 China

**Keywords:** Idiopathic non-obstructive azoospermia, Spermatogonial stem cell, Long non-coding RNA, MALAT1, Spermatogenesis

## Abstract

Metastasis-associated lung adenocarcinoma transcript 1 (MALAT1), is a long non-coding RNA localized in the cell nucleus, known for its multifunctional roles, including potential involvement in spermatogenesis. This study investigates the mechanism by which MALAT1 dysregulation contributes to the pathogenesis of idiopathic non-obstructive azoospermia (iNOA). We analyzed MALAT1 levels in two gene expression profiling datasets comprising patients with obstructive azoospermia (OA) who have normal spermatogenesis and 13 patients with iNOA. The dysregulation of MALAT1 along with the expression levels of its negatively correlated genes were confirmed in a larger cohort of 24 OA patients and 38 iNOA patients. We examined the effects of MALAT1 overexpression in primary human spermatogonial stem cells (SSCs) and Sertoli cells. Additionally, we assessed DNA methylation, as well as levels of H3K27me3 and H3K27Ac level near the etv5 promoter region using ChIP-qPCR. We observed that MALAT1 was overexpressed in testes of iNOA patients with its levels negatively correlating with six spermatogenesis related genes and positively correlated with three others. Overexpression of MALAT1 in SSCs repressed proliferation and induced apoptosis while also suppressing ETS variant transcription factor 5 (ETV5) expression by promoting H3K27 tri-methylation of the ETV5 promoter. Overexpression of MALAT1 in Sertoli cells did not induce apoptosis but impaired their cell supporting function. In conclusion, MALAT1 overexpression in SSCs contributes to the pathogenesis of iNOA via downregulating ETV5 expression and promoting cell apoptosis.

**Supplementary Information:**

The online version contains supplementary material available at 10.1186/s43556-024-00235-6.

## Introduction

Infertility, one of the main health problems, affects 8–12% of couples in the reproductive age worldwide [1].Male factors attributed to 30–50% cases of infertility, yet addressing male infertility due to spermatogenesis defects is significantly more challenging than female infertility [2–5]. Non-obstructive azoospermia (NOA) is one of the most severe forms of male infertility, affecting 10–15% of infertile men [6, 7]. While various factors contribute to NOA, the etiology of more than 70% of cases is still unknown, referred to as idiopathic NOA (iNOA) [8]. Therefore, identifying the causes of iNOA, exploring the underlying mechanisms and developing innovative therapies for iNOA treatment are urgently needed.

Metastasis-associated lung adenocarcinoma transcript 1 (MALAT1) is a long non-coding RNA, which is identified to be overexpressed in multiple types of tumors [9–12]. Although MALAT1 knockout mice do not have significant phenotype, immerging evidence indicates that MALAT1 play a role in female reproductive system [13, 14]. Wang Y et al. reported that dysregulated MALAT1 may be related to disordered crosstalk between embryo and mother and contributes to the pathogenesis of recurrent pregnancy loss [15]. Polymorphism rs591291 C > T in MALAT1 was found to be related to the increased risk of endometriosis [16]. Recently, Afsaneh-Jaberi Asl et al. reported that the level of MALAT1 is reduced in semen samples from infertile men and related to high level of malondialdehyde, increased DNA damage, and reduced motility of sperm [17]. However, it remains unclear whether MALAT1 plays a role during spermiogenesis in testis.

ETS variant transcription factor 5 (ETV5) is a member of the ETS transcription factor family, having a conserved ETS domain that binds to a DNA sequence with a 5′-GGA(A/T)−3′ core [18]. ETV5 is expressed in brain, lung and testis playing a role in various developmental processes [19–21]. In the testis of neonatal mice, ETV5 is found to be expressed in both Sertoli cells (SCs) and germ cells where it controls the renew and differentiation of spermatogonial stem cells (SSCs) [22–24]. Etv5 null mice undergo the first wave of spermatogenesis, but they experience a progressivelydecline in SSCs, which may be the reason of impaired proliferation [22, 25]. However, the role of ETV5 in human infertility is unknown.

In eukaryotes, gene expression is tightly controlled by epigenetic factors including DNA methylation, histone methylation, and histone acylation. Generally, CpG enriched regions in the genome, known as CpG islands, are related to gene promoters, and the methylation of these CpG islands typically leads to gene silencing [26, 27]. Histone lysine methylation on position 27 of H3 (H3K27me) is particularly important for gene silencing by the action of polycomb repressive complex, with the region of which are extensively overlapped with CpG isolands in somatic cells [28, 29]. In contrast, the acetylation of H3K27 contributes to an open chromatin state that facilitates gene expression [30]. In cancer cells, MALAT1 has been shown to modulate H3K27 methylation and controls downstream genes expression. However, its role in spermatogenesis remains unclear.

In this study, we identified the dysregulation of MALAT1 in patients with iNOA by analyzing publicly available datasets. We confirmed the dysregulation of MALAT1 in a larger cohort containing 24 OA patients and 38 iNOA patients. The biological function of MALAT1 was evaluated in primary human SSCs and SCs, and the results indicated that the overexpression of MALAT1 in SSCs repressed proliferation and induced apoptosis. MALAT1 overexpression in SCs impaired cell supporting function. We identified that MALAT1 controlled ETV5 expression by promoting H3K27 tri-methylation of the promoter region. MALAT1 overexpression contributes to the pathogenesis of iNOA via inducing the dysfunction of SSCs and SCs.

## Results

### MALAT1 is overexpressed in testis of patients with iNOA and relates to dysregulation of genes that involved in spermatogenesis

**Fig. 1:**
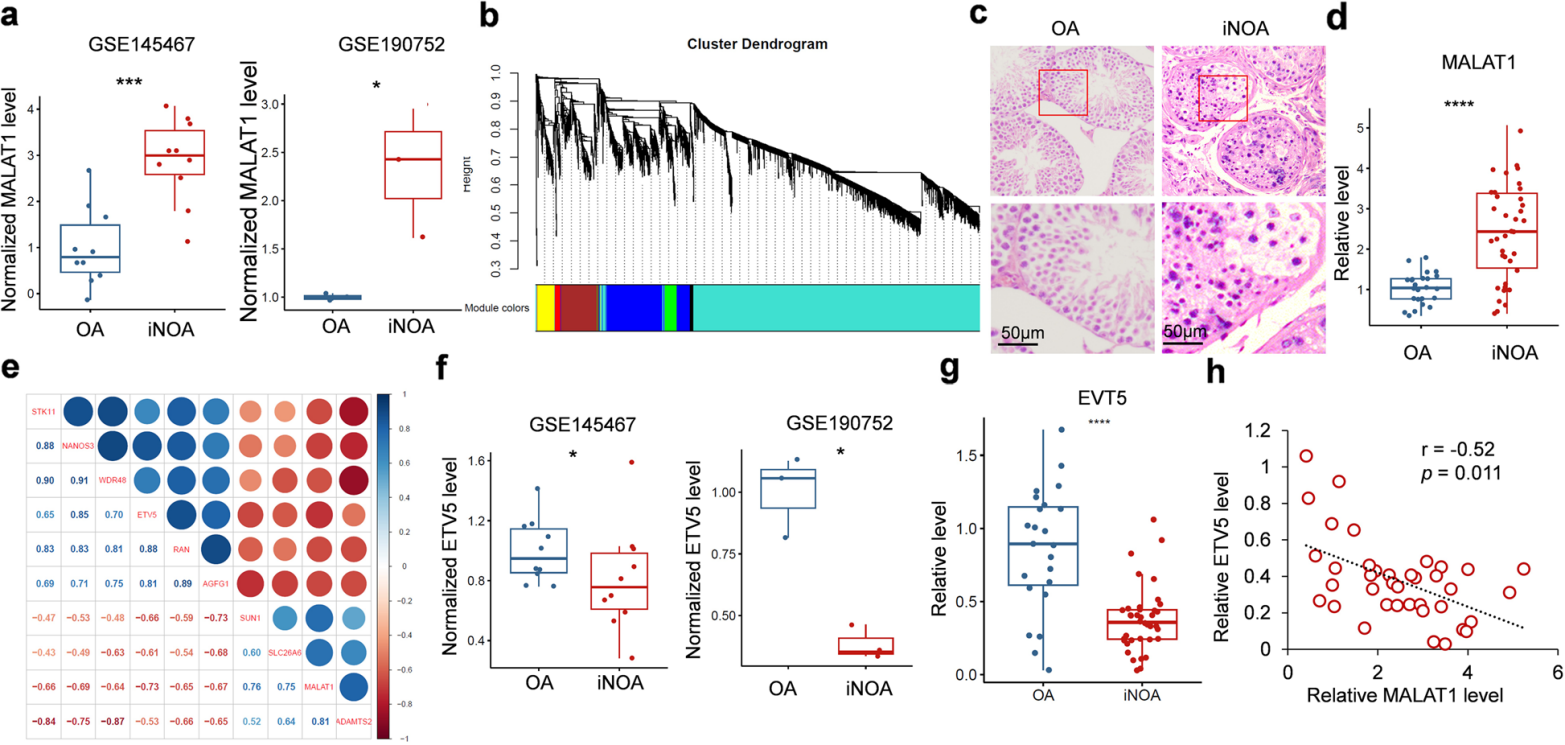
MALAT1 is overexpressed in iNOA testis and related to the dysregulation of genes involved in spermatogenesis. **a** MALAT1 in OA and iNOA testis were analyzed in two reported gene expression profiling datasets (GSE145467 and GSE190752). **b** WGCNA showing co-expressed genes. **c** (A) representative images of OA and iNOA testes. Scale bar = 50 μm. **d** MALAT1 level in OA (*n* = 24) and iNOA(*n* = 38) testis was detected by RT-qPCR. **e** Correlation analysis between MALAT1 and genes functionally involved in spermatogenesis. The 9 significantly correlated genes were shown. **f** ETV5 mRNA level in gene expression profiling datasets (GSE145467 and GSE190752). **g** EVT5 mRNA level in OA (*n* = 24) and iNOA (*n* = 38) testis detected by RT-qPCR. **h** correlation analysis between the levels of MALAT1 and ETV5

In order to identify the roles of MALAT1 in the pathogenesis of iNOA, gene expression profiling datasets, GSE145467(10 iNOA and 10 OA) and GSE190752 (3 iNOA and 3OA), were analyzed. We observed that the MALAT1 level was significantly upregulated in testis of patients with iNOA (Fig. 1a). Weighted Gene Co-Expression Network Analysis (WGCNA) was used to analyze the GSE145467 dataset, and MALAT1 was found to be involved in the core genes related to iNOA phenotype, which labeled as blue module (Fig. 1b, Fig. S1). We subsequently recruited a cohort of 24 patients with OA and 38 patients with iNOA. The presence or absence of germ cells in testicular biopsies from these patients was confirmed through histological analysis (Fig. 1c). We detected the MALAT1 level in testes biopsies and confirmed the upregulation of MALAT1 in patients with iNOA (Fig. 1d).

### MALAT1 is negatively correlated with ETV5 level in testis from patients with iNOA

To further explore whether MALAT1 is functionally related to spermatogenesis, the correlation between MALAT1 and 411 genes that involved in spermatogenesis (GO:0007283) were analyzed.We noticed that the expression of six genes (STK11, NANOS3, WDR48, ETV5, RAN and AGFG1) was negatively correlated with MALAT1 in testis, while the levels of three genes (SUN1, SLC26A6 and ADAMTS2) were positively correlated with MALAT1 (Fig. 1e). Meanwhile, 4 out of 5 MALAT1 negatively correlated genes (STK11, WDR48, ETV5, RAN and AGFG1) were reduced in patients with iNOA of both cohorts (Fig. S2, Fig. 1f, and Fig. S3). Furthermore, ETV5 was confirmed to be downregulated in our recruited patients with iNOA (Fig. 1g), which is negatively correlated with MALAT1 (*r* = −0.52, *p* = 0.011) level in testes (Fig. 1h). Considering ETV5 was the first strongest MALAT1 negatively correlated gene, and its critical roles in spermatogenesis, the following research focused on dissect the relationship between MALAT1 and ETV5.

### MALAT1 represses ETV5 expression and induces apoptosis in SSCs

**Fig. 2:**
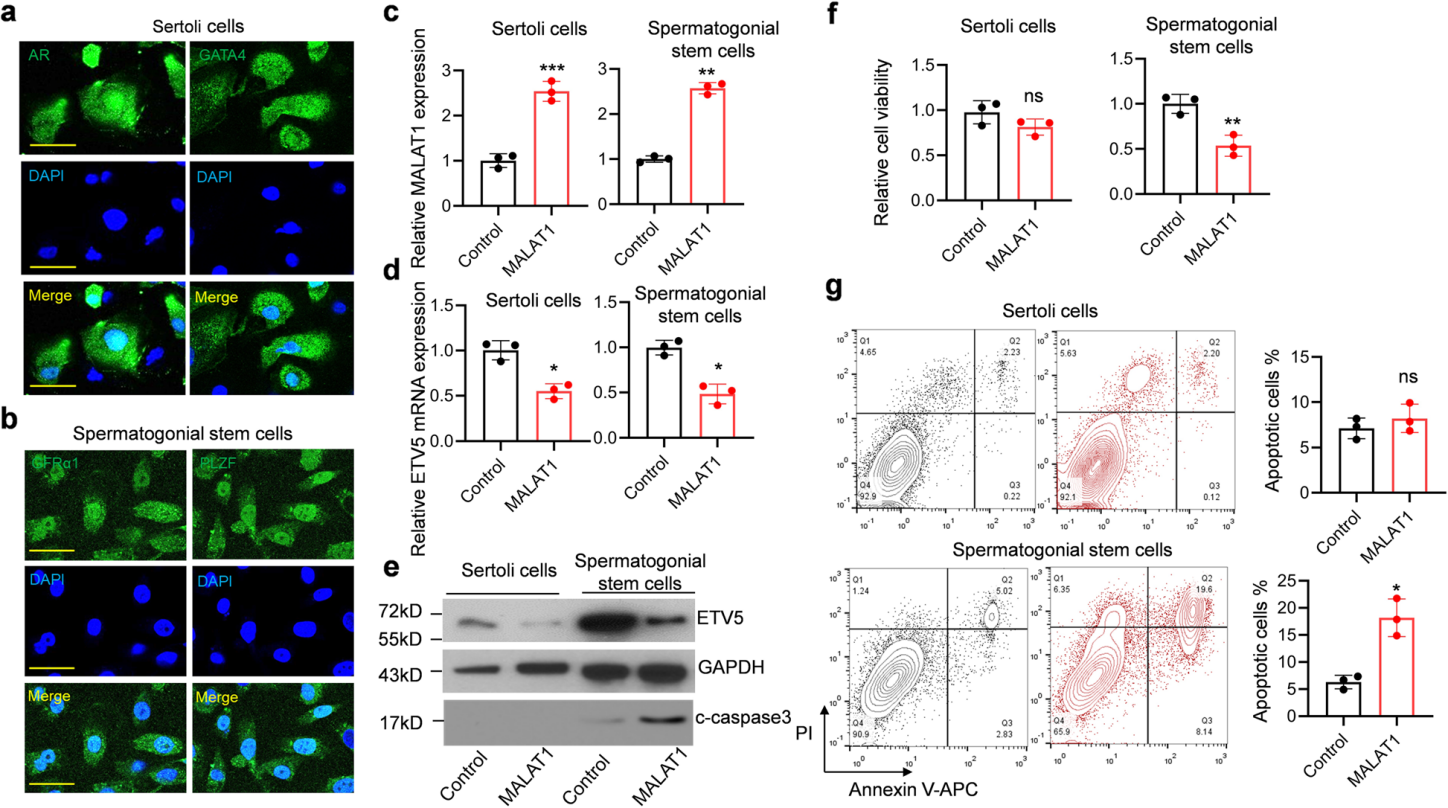
MALAT1 inhibits ETV5 expression and induces SSCs apoptosis. **a** and **b** Primary Sertoli cells and SSCs identification. Immunofluorescence staining AR and GATA4 was used to confirm the Sertoli cell, and GFRα1 and PLZF was used to confirm spermatogonial stem cells. Scale bar = 100 μm. **c **MALAT1 overexpression vector was transfected into human primary SSCs and Stertoli cells. RT-qPCR was used to confirm the upregulation of MALAT1. **d** ETV5 expression was detected by RT-qPCR. **e** Immunoblotting to examine the protein level of ETV5. **f** Cell viability was determined by MTT assay. **g** Apoptotic cells were quantified by flow cytometry

**Fig. 3:**
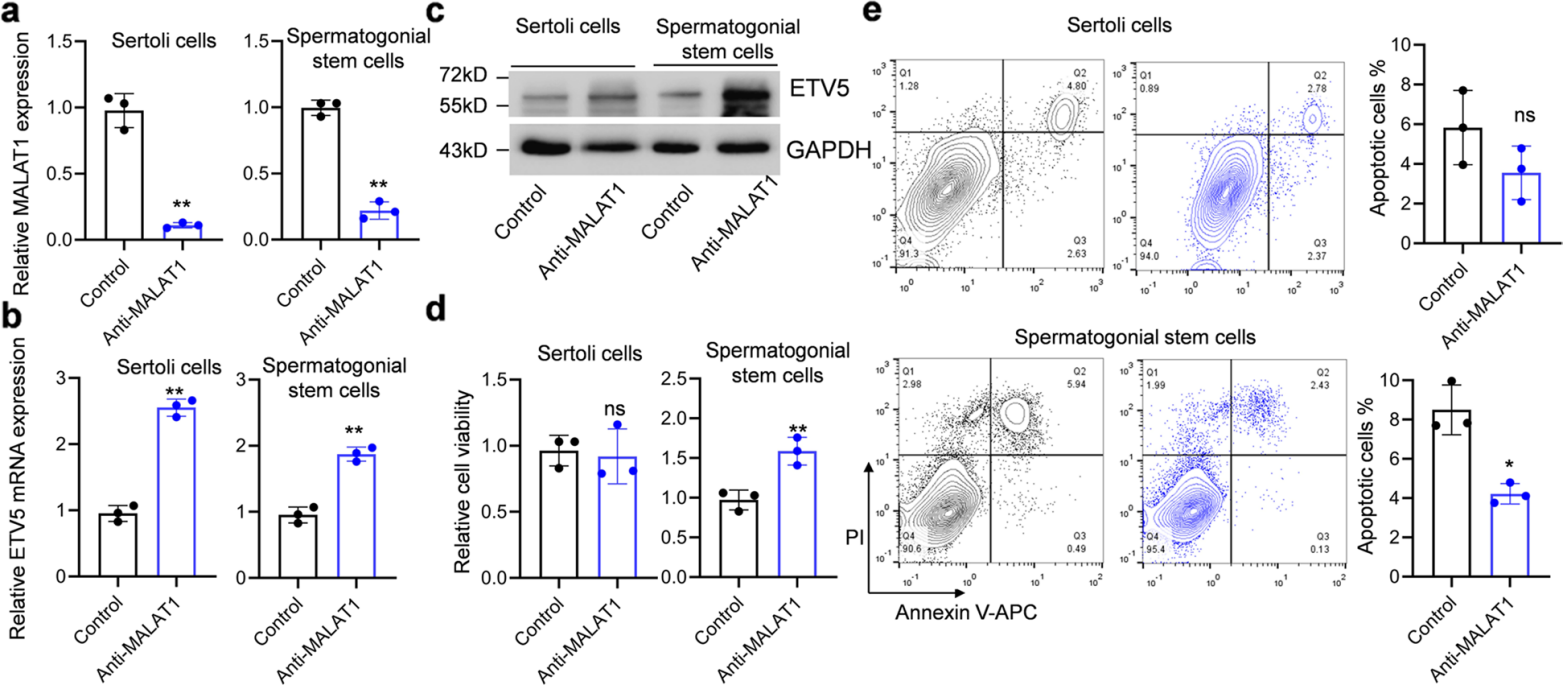
Knockdown MALAT1 in SSCs upregulates ETV5 expression, increased viability and repressed apoptosis. **a** Sertoli cells and SSCs were transfected with gapmer antisense oligonucleotide targeting MALAT1. The levels of MALAT1 were detected by RT-qPCR. **b** The levels of ETV5 mRNA were detected by RT-qPCR. **c** The protein level of ETV5 was examined by immunoblotting. **d** The cell viability was detected by MTT assay. **e** The percentage of apoptotic cells was examined by flow cytometry

To explore the biological function of MALAT1, primary SSCs and SCs were isolated from testis biopsies. Immunofluorescence staining AR and GATA4 was used to confirm the Sertoli cell (Fig. 2a), and GFRα1 and PLZF was used to confirm spermatogonial stem cells (Fig. 2b). The MALAT1 overexpression vector was transfected into cells using electroporation, with an empty vector as the control. A more than 2.5-fold upregulation of MALAT1 was found in both SSCs and SCs 2-days after transfection (Fig. 2c). Meanwhile, the mRNA and protein levels of ETV5 were found reduced in MALAT1 overexpressed cells (Fig. 2d and e). MALAT1 overexpression SSCs had reduced viability and increased apoptosis (Fig. 2f and g). However, MALAT1 overexpressed SCs did not show significant alteration in viability and apoptosis (Fig. 2f and g). Meanwhile, MALAT1 was knocked down in SSCs and SCs (Fig. 3a). The ETV5 expression was upregulated in MALAT1 knockdown cells (Fig. 3b and c). However, only MALAT1 knockdown SSCs exhibited increased viability and reduced apoptosis (Fig. 3d and e). These results indicated that the function of MALAT1 was cell type specific which only induced apoptosis of SSCs via inhibiting ETV5.

### MALAT1 represses ETV5 expression via promoting H3K27 tri-methylation of the promoter region

**Fig. 4:**
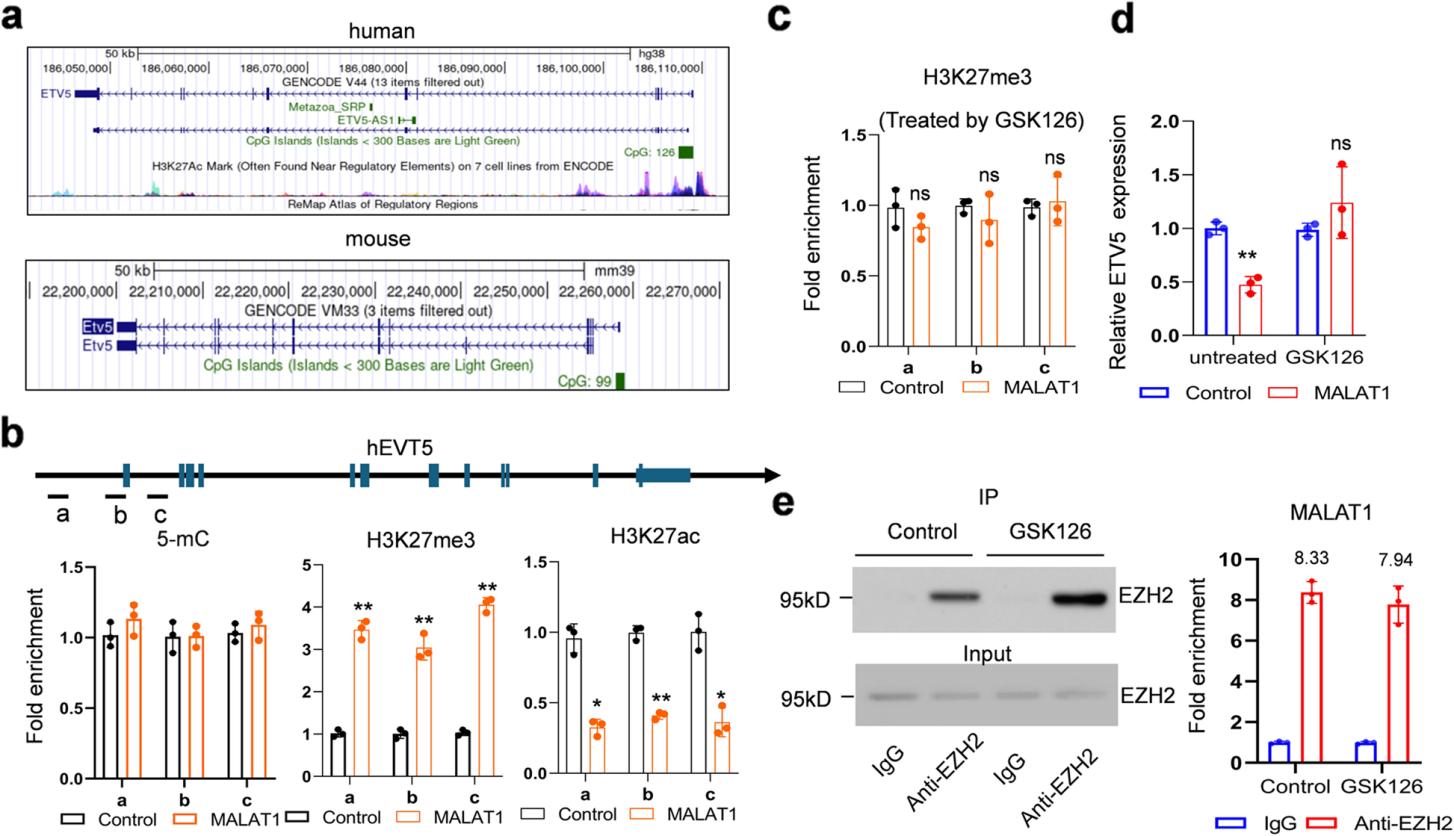
Overexpression of MALAT1 in SSCs promotes methylation and inhibits acetylation of H3K27 of the ETV5 promoter region. **a** Human and mouse *etv5* gene loci information. **b** ChIP-qPCR assay to detect the DNA methylation, H3K27me3 and H3K27Ac levels near the *etv5* promoter region. **c** ChIP-qPCR assay to detect the H3K27me3 levels of the etv5 promoter region in GSK126 treated SSCs. **d** RT-PCR detected ETV5 expression in GSK126 treated or untreated SSCs. **e** RIP assay following RT-qPCR to examine the interaction between MALAT1 and EZH2

MALAT1 has been identified to bind with Enhancer of zeste homolog 2 (EZH2) and control the downstream genes expression through modifying histone methylation in cancer cells [31, 32]. To investigate the mechanism by which MALAT1 controls ETV5 expression, we checked the loci of *etv5* gene in human and mouse genome. A 126 bp CpG island and a 99 bp CpG island were found around the first exon of human and mouse *etv5* gene respectively (Fig. 4a). Meanwhile, the H3K27 can also be acetylated around the first exon of human *etv5 *(Fig. 4a), suggesting that ETV5 expression may be controlled by DNA methylation and chromatin acetylation. Subsequently, ChIP-qPCR assays using antibodies targeting 5-mC, H3K27me3 or H3K27Ac were employed to examine the DNA methylation, H3K27 methylation or H3K27 acetylation. Three pairs of primers were designed to amplify three segments (a, b, and c) of the DNA sequence around the first exon of ETV5 (Fig. 4b). We observed that *etv5* promoter region had increased methylation and reduced acetylation modification in MALAT1 overexpression SSCs, but the DNA methylation status was not significantly altered (Fig. 4b). When treated by GSK126, an inhibitor of EZH2, the H3K27me3 level near the etv5 promoter region (Fig. 4c) and EVT5 expression (Fig. 4d) were not significantly altered, in MALAT1 overexpressed SSCs, suggesting that MALAT regulating histone methylation is EZH2 dependent. RIP assay was used to identify the interaction between EZH2 and MALAT1 in SSCs. We observed that EZH2 was successfully enriched by anti-EZH2 antibody. Meanwhile, MALAT1 was also recruited by anti-EZH2 antibody, which was not inhibited by GSK126. These results indicated that MALAT1 overexpression enhanced H3K27me3 modification near the *etv5* promoter region via binding with EZH2.

**Fig. 5:**
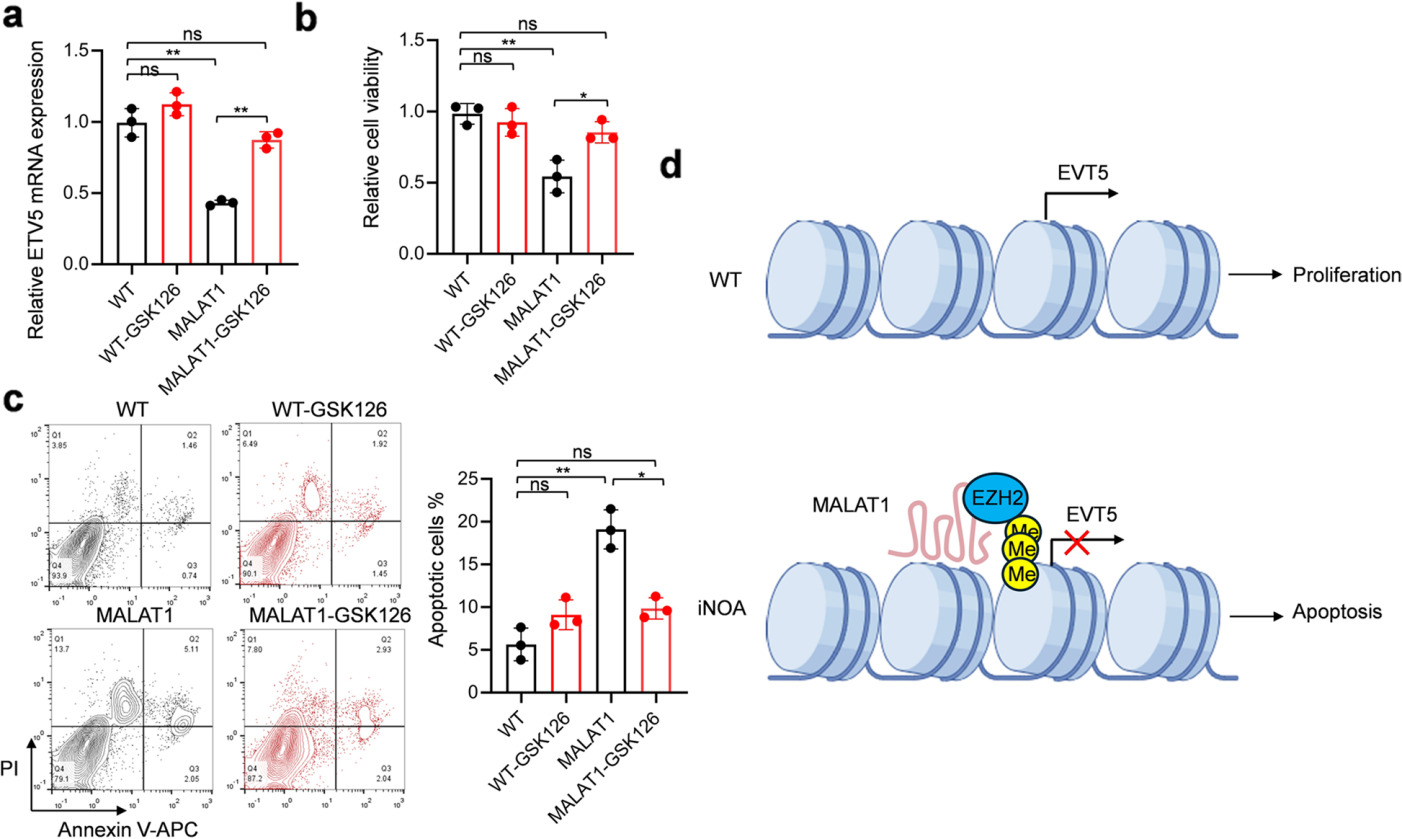
EZH2 inhibition restores the function of MALAT1 in SSCs. SSCs were treated by GSK126 for 48 h. **a** The expression of ETV5 was detected by RT-qPCR. **b** Cell viability was detected by MTT assay. **c** Apoptotic cells were quantified by flow cytometry analysis. **d** The schematic diagram of proposed working model of MALAT in iNOA SSCs

Subsequently, wildtype and MALAT1 overexpressed SSCs were treated with GSK126. GSK126 treatment did not significantly change ETV5 mRNA level in wildtype cells but successfully restored the inhibitory function of MALAT1 on ETV5 expression (Fig. 5a). Similarly, MALAT1 inhibited viability and upregulated apoptosis of SSCs which restored by GSK126 treatment (Fig. 5b and c). As a results, we developed the proposed working model of MALAT1 in iNOA that MALAT1 overexpression in SSCs repressed ETV5 expression by promoting H3K27 tri-methylation which further induced SSCs apoptosis and contributed to iNOA (Fig. 5d).

### MALAT1 overexpression impairs the supporting functions of SCs

**Fig. 6:**
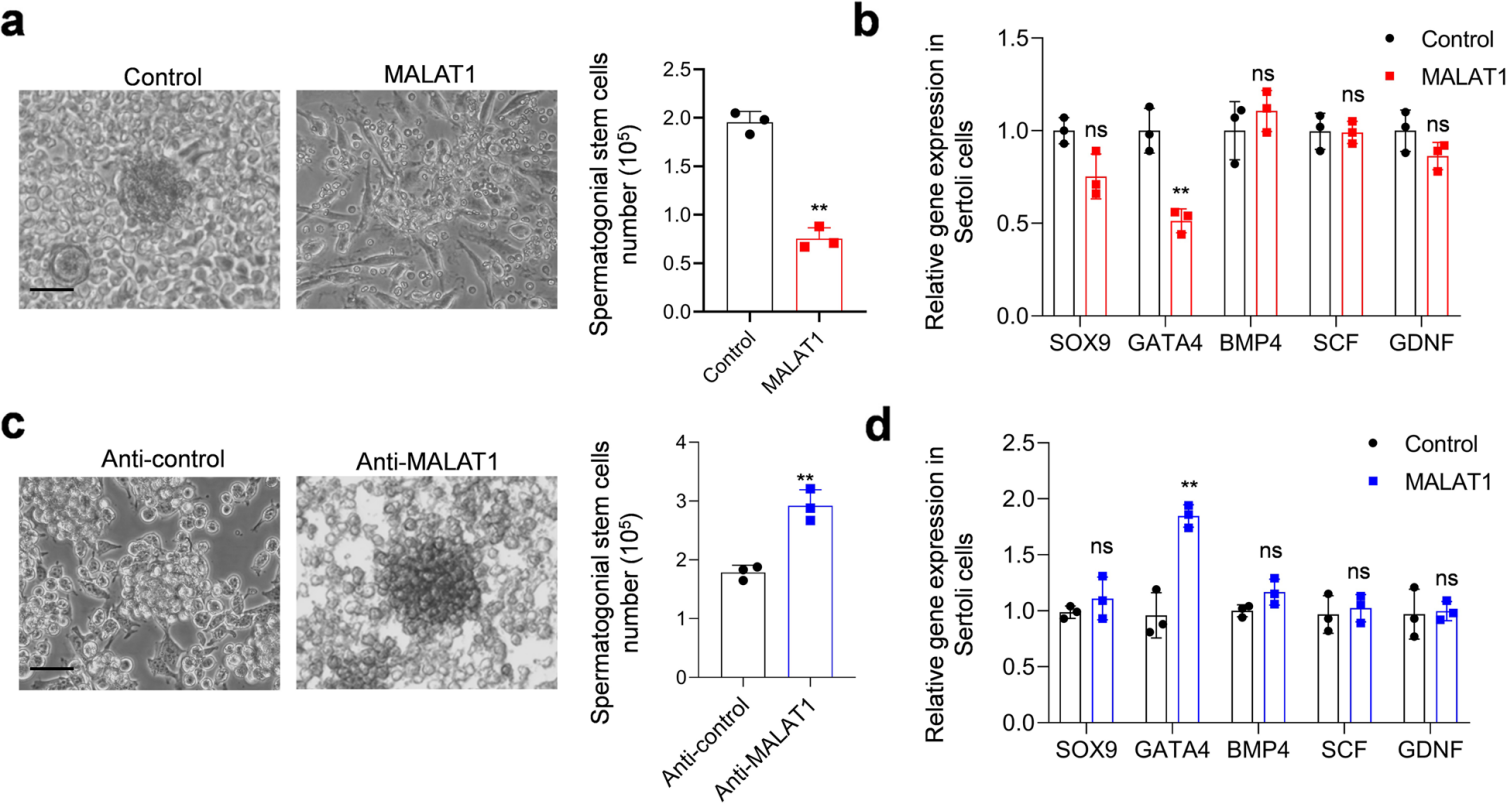
MALAT1 overexpression impaired SCs supporting function. **a** Cell supporting assay. SCs were transfected with control or MALAT1 overexpression vector and then used as feeder cells to support the growth of SSCs. On the 9th day of co-culture, the images of SSCs formed colonies were captured. The number of SSCs were counted after digestion and trypan blue staining. Scale bar = 100 μm. **b** RT-qPCR was employed to detected levels of SOX9, GATA4, BMP4, SCF and GDNF in SSCs after co-culture. **c** Cell supporting assay. SCs were transfected with gapmer antisense oligonucleotide targeting MALAT1 or control short oligonucleotide and then used as feeder cells to support the growth of SSCs. On the 9th day of co-culture, the images of SSCs formed colonies were captured. The number of SSCs were counted after digestion and trypan blue staining. Scale bar = 100 μm. **d** RT-qPCR was employed to detected levels of SOX9, GATA4, BMP4, SCF and GDNF in SSCs after co-culture

Finally, we checked the potential impact of MALAT1 dysregulation on SCs by using in vitro cell supporting assay. SCs were transfected with control or MALAT1 overexpression vector and then used as feeder cells to support the growth of SSCs. The number of SSCs were counted 9 days after co-culture. Five typical genes that expressed in SCs were detected by RT-PCR in different groups of SCs 48 h after transfection. We observed SSCs cultured with MALAT1 overexpression SCs formed smaller colonies, and their counts were less than the control group (Fig. 6a). Meanwhile, the level of GATA4 was reduced in SSCs that cultured with MALAT1 overexpression SCs (Fig. 6b). When SSCs were cultured with MALAT1 knockdown SCs, they formed bigger colonies and had increased proliferation (Fig. 6c). GATA4 expression was upregulated in SSCs cultured with MALAT1 knockdown SCs (Fig. 6d). These results indicated that MALAT1 overexpression impaired the supporting function of SCs.

## Discussion

Dysregulated non-coding RNAs play a role during the pathogenesis of iNOA but the roles of MALAT1 in iNOA remains unclear. Our previous study focused on mitochondria dysfunction in Sertoli cells, and identified the imbalance of mitochondria fission and fusion contribute to the pathogenesis of iNOA [33]. In this study, we analyzed reported gene expression profiling data and found the significant upregulation of MALAT1 in iNOA testis, which is negatively correlated with the expression of 6 and positively correlated with 2 genes that functionally involved in spermatogenesis. We confirmed the negatively correlation between MALAT1 and ETV5 in the same cohort including 24 patients with OA and 38 patients with iNOA and then identified the mechanism by which MALAT1 represses ETV5 expression in SSCs. To our knowledge, this is the first report demonstrating the possible role of MALAT1 in iNOA and provide a potential target for iNOA treatment.

Spermatogenesis is marked by robust cell division and specialization that continues throughout a person’s lifetime, which is accompanied by the establishment, replication, and passing down of epigenetic modifications, specifically methylation of the histone H3K27 protein. Mu et al.‘s research demonstrated that the Polycomb Repressive Complex 2 (PRC2) plays a critical role in maintaining spermatogonial stem cells and facilitating meiotic progression by repressing the expression of genes that are specific to somatic cell stages as well as meiotic cell stages, using the H3K27me3 epigenetic mark [34]. MALAT1 was identified to interact with EZH2, the main methyltransferases of PRC2, and modulate downstream genes expression in multiple kinds of cancer cells [35–37]. In this study, we identified the MALAT1 inhibited ETV5 expression in SSCs through upregulating the tri-methylation of H3K27 near the promoter region. EZH2 inhibitor treatment could restore the function of overexpressed MALAT1, indicating that MALAT1 may guide EZH2 to ETV5 promoter region, inhibit ETV5 transcription and induce SSCs apoptosis.

MALAT1 is a multiple functional long non-coding RNA whose function may be cell type specific. Initially, MALAT1 was identified to be an oncogene promoting tumor cell proliferation, metastasis and inhibiting apoptosis. However, emerging evidence indicates that the function of MALAT1 varies in different types of cells. Jongchan Kim et al. constructed MALAT1 transgenic mice in 2018 [38] and observed that MALAT1 overexpression suppressed breast cancer metastasis. C Cheng et al. reported that overexpression of MALAT1 attenuated viability and induced apoptosis in oxidized low-density lipoprotein treated vascular smooth muscle cells [39]. Likun Ma group identified that MALAT1 is overexpressed in acute myocardial infarction mouse model which upregulates PTEN and promotes myocardial apoptosis by sponging miR-320 [40]. In this study, we observed that MALAT1 overexpression in SSCs inhibits the viability and promotes apoptosis. However, these phenotypes were not observed in SCs. This inconsistency may be the result of the differentially expressed proteins and miRNAs in these two different types of cells. Herein, we identified that MALAT1 induces apoptosis by regulating ETV5 expression but the function of MALAT1 in SCs needs to be further unveiled.

MALAT1 is a cell nucleus localized lncRNA which is found to be expressed in mouse testis. MALAT1-knockout mice are normal in fertility [14], although the expression of MALAT1 neighboring genes appeared to be affected. Recently, Afsaneh-Jaberi Asl et al. reported that the level of MALAT1 is reduced in semen samples from infertile men and related to high level of malondialdehyde, increased DNA damage, and reduced motility of sperm [17]. They proposed that downregulation of MALAT1 may be related to its antioxidant function. However, they just constructed the correlation between dysregulated MALAT1 and abnormal sperm phenotype. Much less is known about the role of MALAT1 in regulating human spermatogenesis. In this study, we identified that MALAT1 is overexpressed in the testis tissue samples from patients with iNOA who have impaired spermatogenesis. This inconsistency may be the result of different sample type, and the cell type specific function of MALAT1. Our following functional study in primary human SSCs and Sertoli cells indicated that MALAT1 overexpression induces apoptosis in SSCs without changing Sertoli cells’ function which partially unveiled the function of MALAT1 in spermatogenesis in human.

There are limits of the present study. First, through bioinformatics analysis, MALAT1 was found to be related to the expression of 9 spermatogenesis related genes suggesting the multifunctional role of MALAT1. This research only focused on the MALAT1/ETV5 axis and the full function of MALAT1 during spermatogenesis needs to be further investigated. Second, Sertoli cells support and provide the nutrient for SSCs. We overexpressed MALAT1 in SCs but did not observe altered proliferation and apoptosis. Through cell supporting assay, we observed that MALAT1 overexpression impaired the supporting function of SCs. However, the underlying mechanism needs to be further unveiled.

Collectively, this study dissected the function of MALAT1 using primary human SCs and SSCs to investigate the roles of MALAT1 in the pathogenesis of iNOA. We identified that MALAT1 overexpression in SSCs could repressed ETV5 expression through inducing histone hypermethylation by binding with EZH2 and induced apoptosis. Furthermore, upregulation of MALAT1 in SCs impaired the supporting function of SCs. These findings raise the possibility that targeting MALAT1 could be an attractive avenue for the therapeutic intervention of iNOA.

## Materials and methods

### Cohorts

The experiments conducted in this study received approval from the Ethics Committee of the Second Affiliated Hospital of Zhengzhou University (approval no. KY2024151). The study involved 24 patients with obstructive azoospermia (OA) who exhibited normal spermatogenesis, alongside 38 patients with idiopathic non-obstructive azoospermia (iNOA). Eligible participants had a history of infertility lasting at least one year and were confirmed to have azoospermia through at least three consecutive semen analyses. Histological analysis was used to verify whether germ cells present or absent in the biopsies. All participants were assessed for additional infertility risk factors, which included chromosomal abnormalities, cryptorchidism, orchitis and epididymitis that induced by infections, previous radiotherapy and chemotherapy, undescended testis, hypogonadism, alcohol consumption, abnormal liver function, smoking, and sexually transmitted infections. Azoospermia was classified by the urological team as either obstructive or non-obstructive. Idiopathic non-obstructive azoospermia was defined as non-obstructive azoospermia without a history of cryptorchidism, genetic causes, or other identifiable factors. Among the 24 OA cases, 21 were diagnosed with stones or calcifications in the ejaculatory duct, while 3 cases involved midline cysts of the prostate. The cohort was the same as those in the previous study [33].

For the collection of human testis samples, all participants gave informed consent after receiving thorough information about the study’s objectives and details. Fresh testicular tissues were collected from 5 donors who underwent testicular biopsy or partial excision for various indications: one case involved the contralateral testis following testicular torsion, three cases were related to benign testicular masses, and one case involved the contralateral testis associated with cryptorchidism. These tissues were used to isolate primary spermatogonial stem cells (SSCs) and Sertoli cells. Additionally, 24 samples from patients with obstructive azoospermia (OA) and 38 samples from those with idiopathic non-obstructive azoospermia (iNOA) were collected from excess tissue after testicular sperm extraction procedures. All patients with OA demonstrated normal karyotypes, genotypes, sex hormone levels, and seminiferous tubule morphology appropriate for their age. Table 1 lists the clinical features of patients with OA and iNOA in detail.

**Table 1:** Clinical characteristics of participants [33]

	iNOA (*n* = 38)	OA (*n* = 24)	*P* value
Age (year)	34.1 ± 5.9	32.0 ± 4.9	0.13
BMI(Kg/m^2^)	24.1 ± 2.3	23.9 ± 1.8	0.71
Infertility duration(year)	4.9 ± 1.6	5.2 ± 1.5	0.45
Testicular volume (mL)	12.0 ± 3.2	11.8 ± 2.5	0.77
FSH (IU/L)	13.4 ± 8.3	8.2 ± 6.2	0.084
Testosterone (nmol/L)	13.4 ± 5.7	12.1 ± 6.5	0.40
Inhibin B (pg/mL)	134.2 ± 66.7	168.8 ± 70.3	0.060
LH (mIU/mL)	5.6 ± 2.4	4.7 ± 1.1	0.074
Prolactin (ng/mL)	11.1 ± 3.2	12.3 ± 2.7	0.13
E2 (pg/mL)	22.9 ± 5.2	21.1 ± 2.8	0.073

*FSH* follicle stimulating hormone, *BMI* body mass index, *LH* Luteinizing hormone, *E2* 17β-estradiol

### Primary SCs isolation and identification

We isolated human primary SCs from testicular samples using a previously described two-step digestion method [33]. In brief, seminiferous tubules were separated after an initial digestion with collagenase IV (2 mg/ml) (Gibco) and DNase I (1 µg/ml) (Roche) in 34 °C for 15 min. The seminiferous tubules were then further digested with a mixture of enzymes containing collagenase IV (4 mg/ml), hyaluronidase (2.5 mg/ml) (Sigma), trypsin (2 mg/ml) (Sigma) and DNase I (1 µg/ml). After being seeded into polystyrene-treated dishes containing DMEM/F-12 supplemented with 10% FBS, the cell suspension was cultured for one day at 34 °C in a 5% CO_2_ environment for 1 day. After incubation, the adherent cells were SCs, and the suspended male germ cells and other types of cells were eliminated.

### Primary SSCs isolation

Human primary SSCs were isolated using a two-step enzyme digestion method as described by Chen W. et al. [41]. Testicular samples from patients with OA were initially digested with 2 mg/ml collagenase IV (Gibco) and 1 µg/ml DNase I (Roche) to isolate seminiferous tubules. Seminiferous tubules were then further digested with a mixture of collagenase IV(4 mg/mL), hyaluronidase (2.5 mg/mL), trypsin(2 mg/mL) and DNase I(1 µg/ml) to generate single cells. SSCs were subsequently purified through positive selection using anti-GFRA1 antibody (ab8026, Abcam) coated magnetic beads (Miltenyi Biotec, Germany).

### Datasets collection and analysis

Expression profiling datasets GSE145467 and GSE190752 were downloaded from the Gene Expression Omnibus (https://www.ncbi.nlm.nih.gov/geo) portal. R (version 4.2.2) was used for data analysis through RStudio (Desktop version, 2022.12.0 + 353). A total of 648 genes known to be functionally related to spermatogenesis were selected as candidate genes.

### Hematoxylin and eosin staining

After an overnight fixation in a 4% paraformaldehyde solution, the testicular tissues were embedded in paraffin and sectioned to a 5 μm thickness. After being dewaxed in xylene for 30 min, the paraffin sections were rehydrated for 10 min at each of the following ethanol concentrations: 95, 90, 85, and 70%. Hematoxylin and eosin staining was then applied to the sections. Microscopic pictures were captured to investigate the structural alterations in testicular tissues.

### RNA extraction

Trizol (Invitrogen, Carlsbad, CA, USA) was used to extract total RNAs from samples according to the manufacturer’s instructions. RNA sample’s concentration and purity were assessed using an ND-1000 spectrophotometer (Nanodrop Technologies, Wilmington, DE, USA). Only samples with absorbance ratios of approximately 2.0 at 260 nm/280 nm, and between 1.9 and 2.2 at 260 nm/230 nm were included in the study.

### Quantitative RT-PCR(RT-qPCR)

GAPDH was used as the loading control in RT-qPCR with SYBR Green Real-Time PCR Master Mix (Thermal Fisher Scientific) to measure the amounts of candidate genes. The experiment was carried out at least three times, with each sample in each group being measured in triplicate. The relative levels of target genes were determined using the-ΔΔct method.

### Immunoblotting

Proteins were extracted from cells using RIPA buffer and separated by 10% polyacrylamide gels (Thermo Fisher Scientific), followed by electroblotting onto PVDF membranes (Millipore). After blocking the membranes with 5% non-fat milk, one of the primary antibodies was added and the membranes were incubated for the entire night at 4 °C. After washing with TBST (20 mM Tris, 150 mM NaCl, 0.1% Tween-20, pH = 7.4), the membranes were incubated with HRP-linked secondary antibody. Chemiluminescent detection was carried out using a SuperSignal West Femto Maximum Sensitivity Substrate kit (Thermo Fisher Scientific), with GAPDH as a loading control.

### Cell viability analysis

The cells were plated at a density of 2 × 10^4^ cells per well in a 96-well plate and were grown overnight at 37 °C with 5% CO_2_ in DMEM-F12 medium. CellTiter 96^®^ AQueous One Solution Reagent (20 µl) were added to each well, and the plate was incubated for an additional 4 hours at 37 °C in a humidified environment with 5% CO_2_.The absorbance at 490 nm was measured using a 96-well plate reader.

### Flow cytometry analysis

Cells were stained with APC labeled Annexin V and propidium iodide (PI) (BioLegend), following the manufacturer’s protocol. In order to identify apoptotic cells, stained cells were further analyzed using flow cytometry. Flowjo v10.8.1 (Ashland) software was employed to analyze the data.

### Chromatin immunoprecipitation (ChIP) assay

After 5 min of cross-linking with 1% formaldehyde at 37 °C, the cells (1 × 10^6^) were lysed on ice in lysis buffer (1% SDS, 10 mM EDTA, 50 mM Tris-HCl, pH 8.1) for 15 min. The cell lysates were sonicated 10 times and then were centrifuged for 10 min at 12,000 g. The non-specific binding proteins were eliminated by incubating the supernatant with Protein-A/G Sepharose for 1 h at 4 °C. One of the primary antibodies [Anti-5-methylcytosine (5-mC) (ab10805, Abcam), anti-H3K27me3(ab6002, Abcam), H3K27Ac (ab4729, Abcam)], or IgG was then incubated with the precleared chromatin for 4 h at 4 °C. After washing three times with wash buffer (0.25 M LiCl, 1% Triton X-100, 1% deoxycholic acid, 1 mM EDTA, 10 mM Tris-HCl, pH 8.1), the beads were processed for DNA extraction. QPCR was used to assess the enrichment of specific DNA segments.

### Ribonucleoprotein Immunoprecipitation (RIP) assay

Cells were irradiated with 150 mJ/cm2 at 254 nm using a UV cross-linker and then subjected to protein extraction. EZH2 antibody coated beads was used to recruit EZH2 and EZH2 binding RNAs via incubation with the cell lysates at 4 °C for 12 h. After six rounds of washing, the beads were subjected to RNA extraction. RT-qPCR was used to examine the enrichment of MALAT1.

### Cell supporting assay

SCs were transfected with MALAT1 overexpression vector or gapmer antisense oligonucleotide targeting MALAT1. Twenty-four hours after transfection, SSCs were seeded in each well at a density of 1 × 10^5^ cells per well and cultured for 9 days. After being digested for two minutes by 0.25% pancreatin, SSCs were carefully aspirated. The digested cells were culture for another 1 h to remove the potential SCs and then subjected to SSCs counting after staining by trypan blue.

### Statistical analysis

SPSS Statistical version 16 (SPSS Inc., Chicago, IL, USA) was used to analyze the data. A two-tailed Student’s t-test was employed to assess statistical significance between the two comparison groups. One-way ANOVA was used for analysis among multiple groups. Results were deemed significant when *p*-value was less than 0.05.

## Supplementary Information

Supplementary Material 1.

Supplementary Material 2. 

## Data Availability

The data underlying this article are available in the article and in its online supplementary material.
