# Supplementary figures and images for: Metastasis-associated lung adenocarcinoma transcript 1 overexpression in testis contributes to idiopathic non-obstructive azoospermia via repressing ETS variant transcription factor 5
Source: Mol Biomed. 2024 Dec 17;5:71. doi: 10.1186/s43556-024-00235-6 (PMC11649603; doi:10.1186/s43556-024-00235-6)

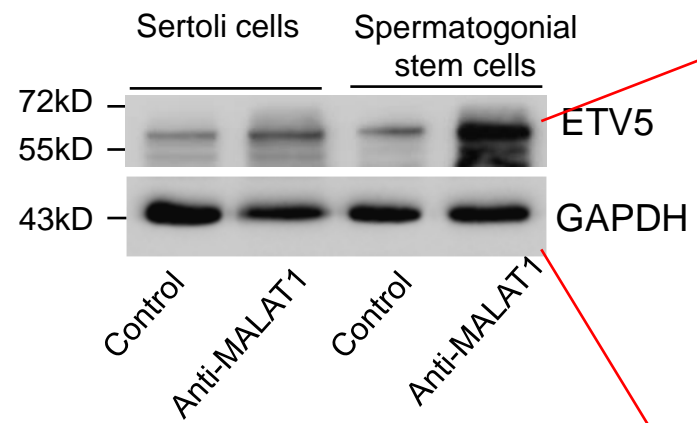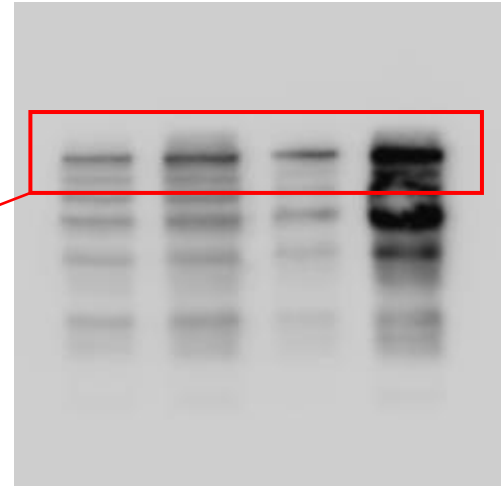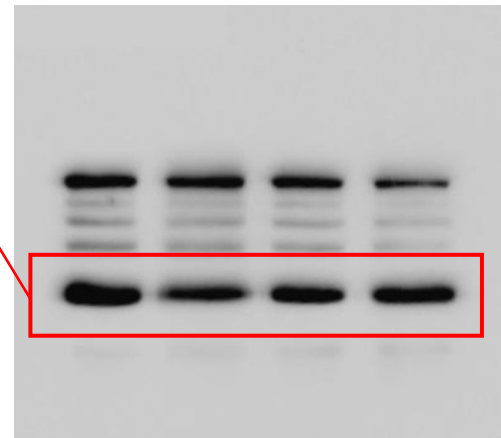

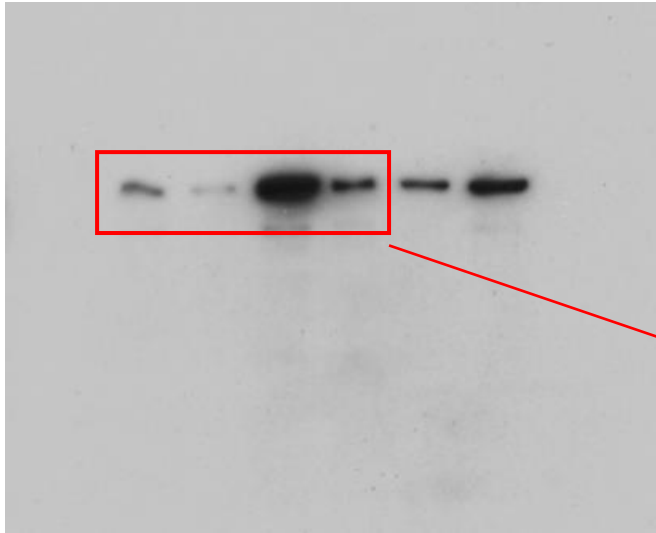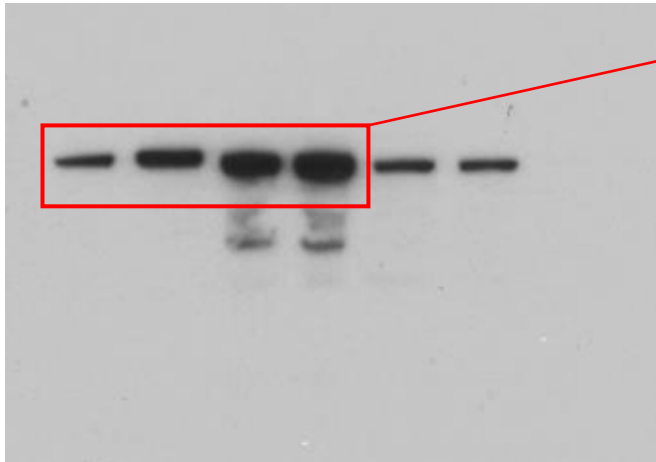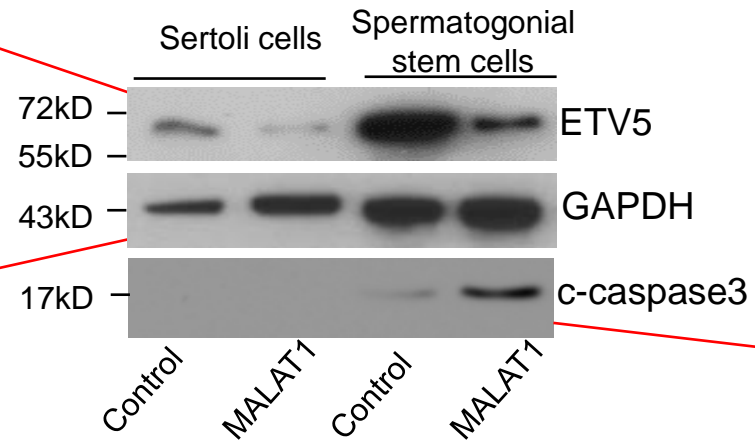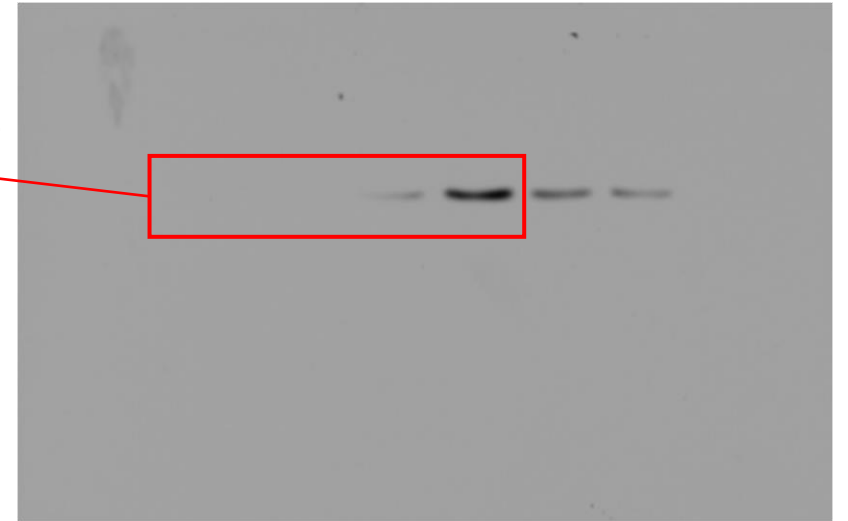

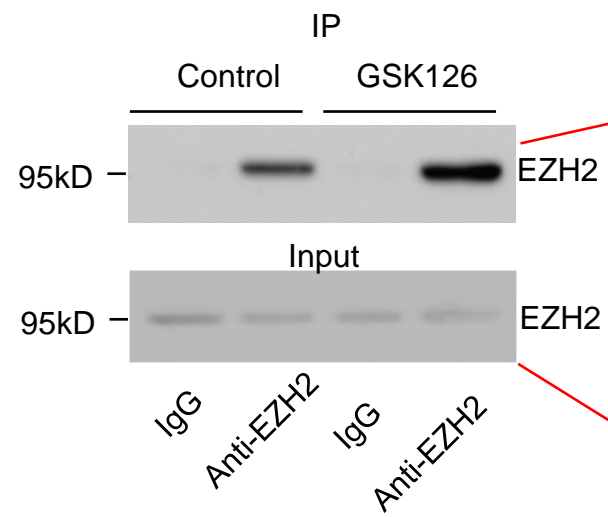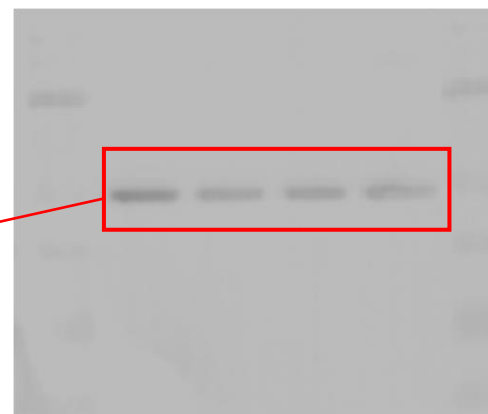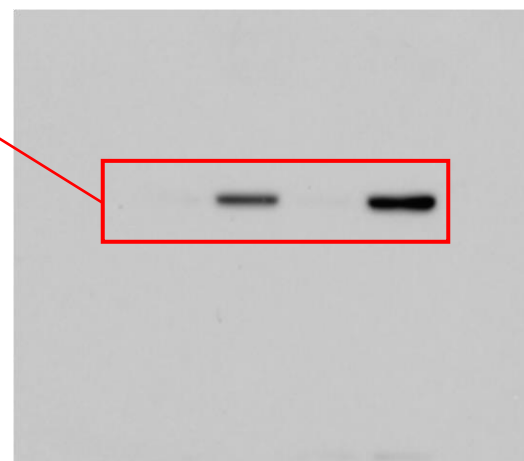

Supplement: Supplementary file 1 — Supplementary Material 1. [file 43556_2024_235_MOESM1_ESM.pdf]
