# Supplementary material for: Metastasis-associated lung adenocarcinoma transcript 1 overexpression in testis contributes to idiopathic non-obstructive azoospermia via repressing ETS variant transcription factor 5
Source: Mol Biomed. 2024 Dec 17;5:71. doi: 10.1186/s43556-024-00235-6 (PMC11649603; doi:10.1186/s43556-024-00235-6)

**Fig.S1. Module-trait association.**

Each row corresponds to a module; each column corresponds to a trait. Each cell contains the test statistic value and its corresponding p value from the linear mixed-effects model.

**Fig. S2. MALAT1 and its correlated genes expression in OA and iNOA tissues.**

The levels of MALAT1 and 9 candidate genes that positively or negatively correlated with MALAT1 were shown.

**Fig. S3. Volcano plots showing the expression of MALAT1 and candidate spermatogenesis related genes.**

**Ethics approval**


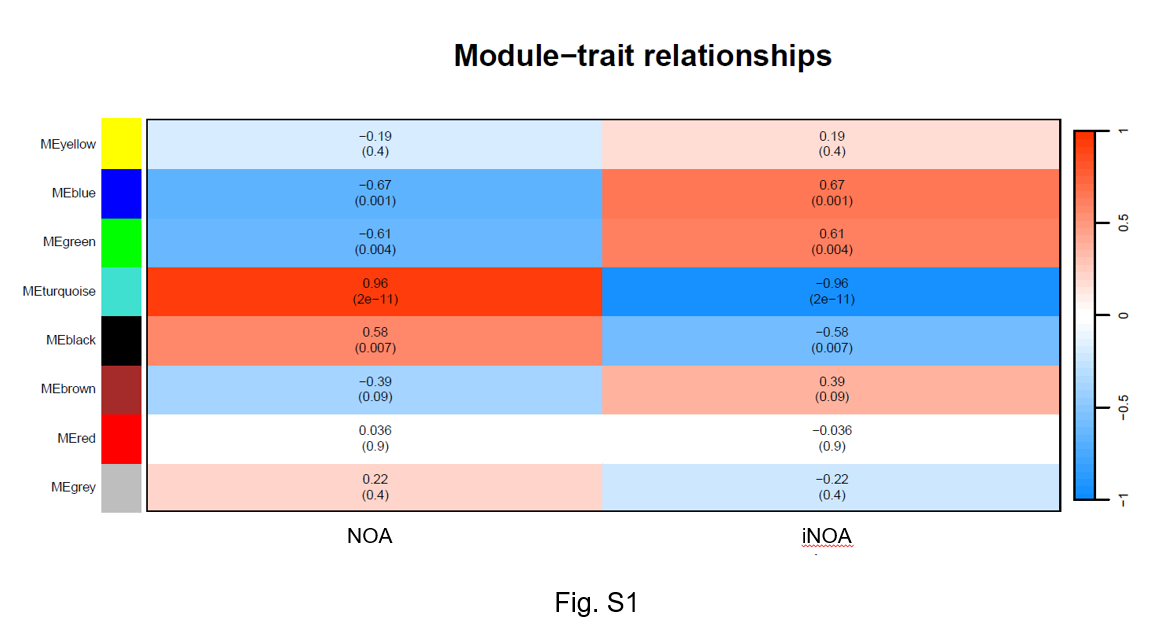


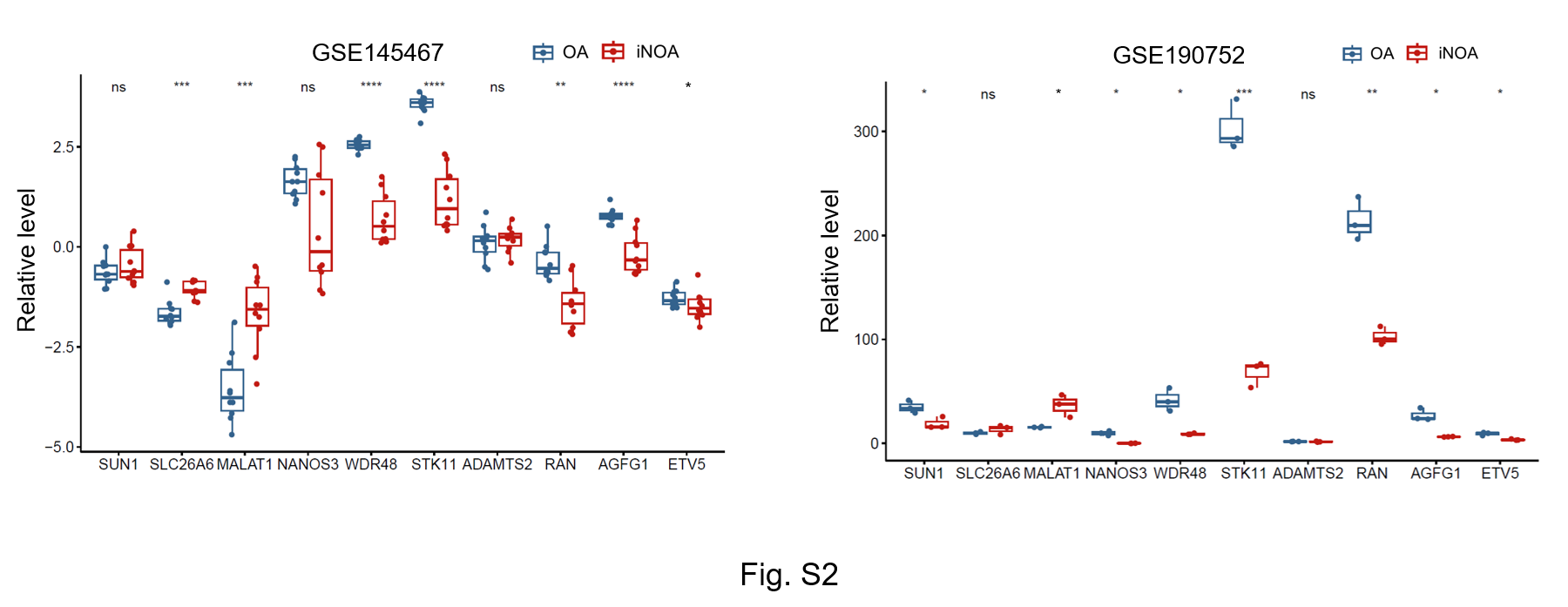


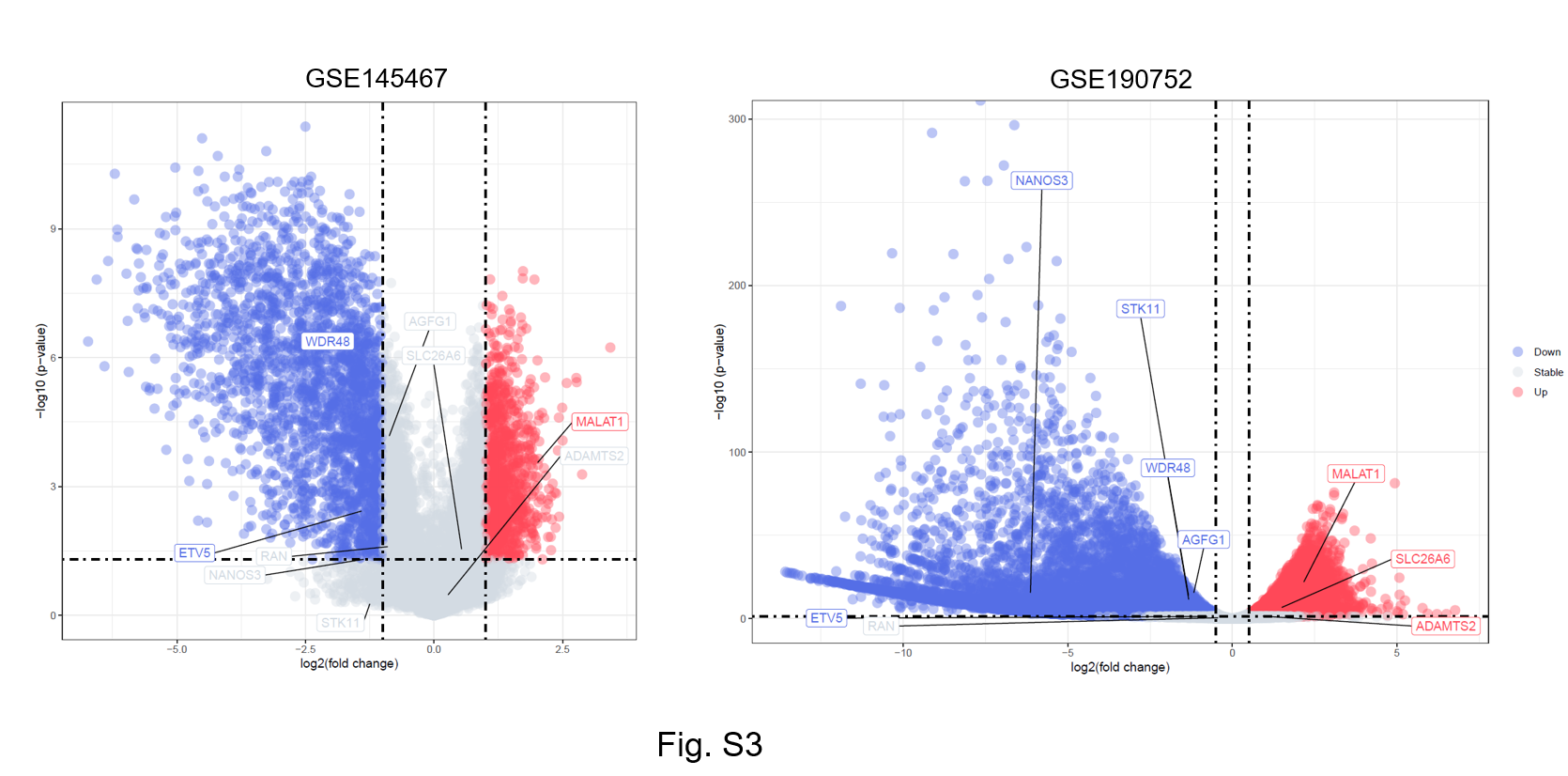


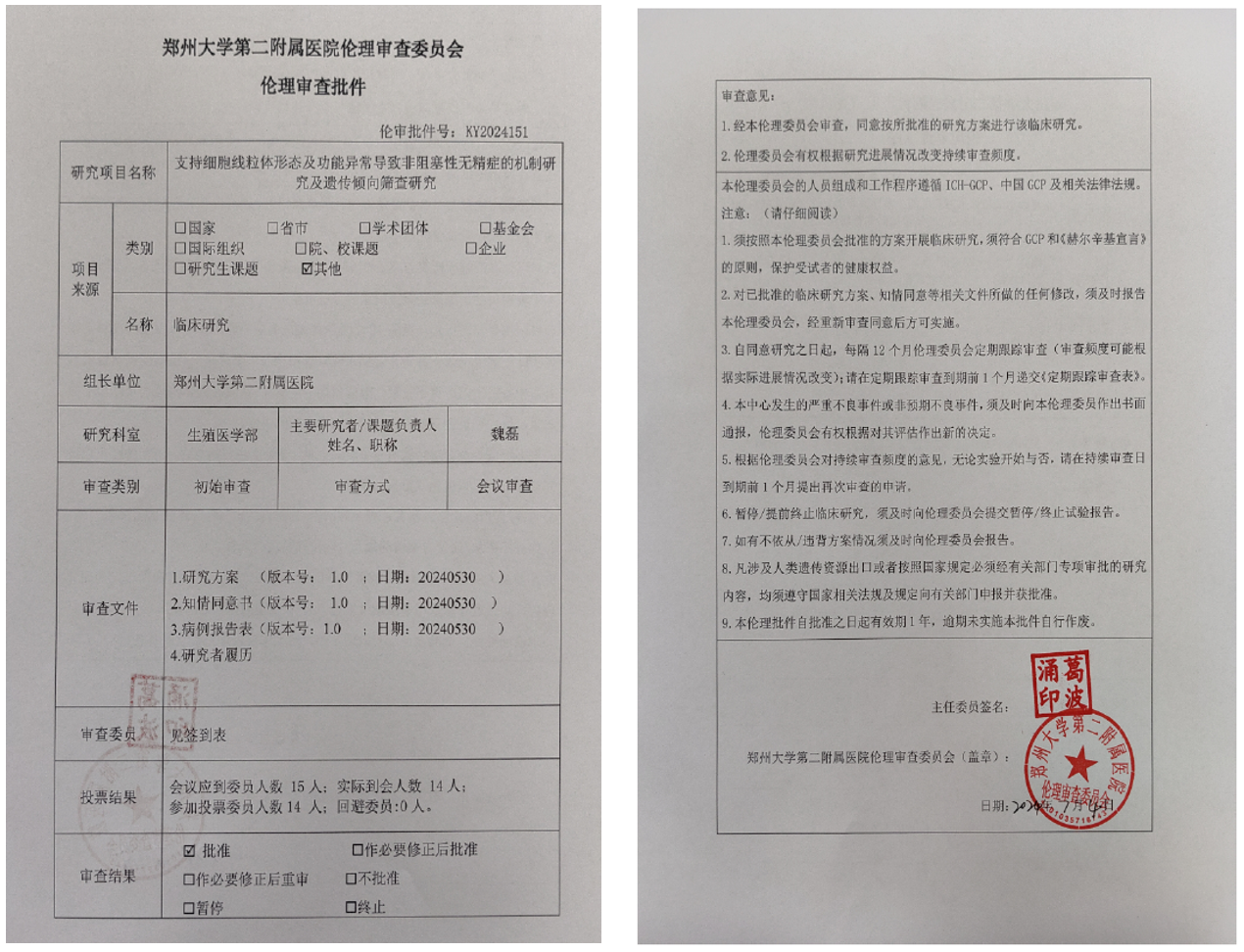

Supplement: Supplementary file 2 — Supplementary Material 2. [file 43556_2024_235_MOESM2_ESM.docx]
